# Emergent Cesarean Delivery in a Patient With Freeman-Sheldon Syndrome Complicated by Preeclampsia, Acute Pulmonary Embolism, and Pulmonary Edema: A Case Report

**DOI:** 10.7759/cureus.20802

**Published:** 2021-12-29

**Authors:** Mohamed Fayed, Mark A Giska, Rebekah C Shievitz, Ami Attali, Joshua Younger

**Affiliations:** 1 Anesthesiology and Perioperative Medicine, Henry Ford Health System, Detroit, USA; 2 Anesthesiology, Perioperative Medicine and Pain Management, Henry Ford Health System, Detroit, USA; 3 Anesthesia, Ascension Providence Hospital, Bingham Farms, USA; 4 Obstetrical Anesthesiology, Henry Ford Health System, Detroit, USA

**Keywords:** freeman-sheldon syndrome, bilevel positive airway pressure, whistling face syndrome, cranio-carpal-tarsal dysplasia, windmill-vane-hand syndrome, syndromic scoliosis, c-section, difficult airway management, acute pulmonary embolism, pre-eclampsia

## Abstract

Freeman-Sheldon syndrome (FSS) is an exceedingly rare congenital disorder with an unspecified prevalence. FSS is caused by a mutation in the embryonic skeletal muscle myosin heavy chain 3 gene. Patients may have facial abnormalities that put them at risk of difficult airway intubation. These facial abnormalities include micrognathia, macroglossia, high-arched palate, prominent forehead, and mid-face hypoplasia. Additionally, skeletal abnormalities such as joint contractures, scoliosis with resultant restrictive lung disease, and camptodactyly (bent fingers) can be noted. These features played an important role in the anesthetic management of our FSS patient. Perioperative planning and optimization were crucial in her anesthetic management as she underwent an urgent cesarean section due to preeclampsia with severe features.

## Introduction

Freeman-Sheldon syndrome (FSS) is an exceedingly rare congenital disorder with an unspecified prevalence, suspected to be caused by a mutation in the embryonic skeletal muscle myosin heavy chain 3 (MYH3) gene. It is thought to be inherited primarily by autosomal or X-linked recessive genes [1]. First described in 1938, it is characterized by facial and skeletal muscle abnormalities [2]. FSS is also referred to as cranio-carpal-tarsal dysplasia, windmill-vane-hand syndrome, and whistling face syndrome due to features of microstomia and pursed lips [3]. Additional facial abnormalities include micrognathia, macroglossia, high-arched palate, prominent forehead, and mid-face hypoplasia. Skeletal abnormalities include joint contractures, scoliosis, ulnar deviation of the fingers, camptodactyly (bent fingers), and talipes equinovarus (club foot). These features play an important role in the anesthetic management of FSS patients. Anesthesiologists should anticipate the possibility of a difficult airway and make the necessary preparations for successful airway management [4]. In addition to the physical characteristics of FSS, there are also metabolic considerations. Multiple case reports have delineated malignant hyperthermia as an outcome of the use of succinylcholine, volatile anesthetic, and other triggering agents [5-9].

To date, most case reports have focussed on the pediatric population. There are limited reports in the literature on adult FSS patients, with only one obstetric case report published in 2016. In this case report, the authors used a combined spinal-epidural technique for an elective cesarean delivery in a parturient with FSS [10]. Our case is unique because our patient had extensive Harrington rods, an acute pulmonary embolus with recent anticoagulation, and severe pre-eclampsia that required an emergency cesarean delivery at less than 28 weeks of gestation.

## Case presentation

**Figure 1:**
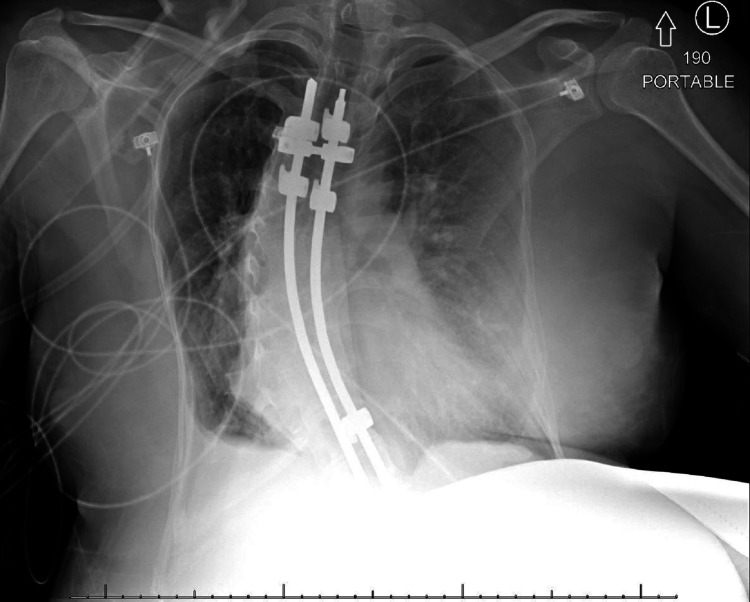
Chest X-ray.

A 34-year-old G1P0 female at 27 weeks and 4 days of gestation presented with shortness of breath. Her medical history included FSS, scoliosis, and restrictive lung disease secondary to scoliosis. Her surgical history was significant for extensive thoracolumbar Harrington rods (Figure 1). Her family history was also significant for a twin with FSS, who had peripartum cardiomyopathy.

**Figure 2:**
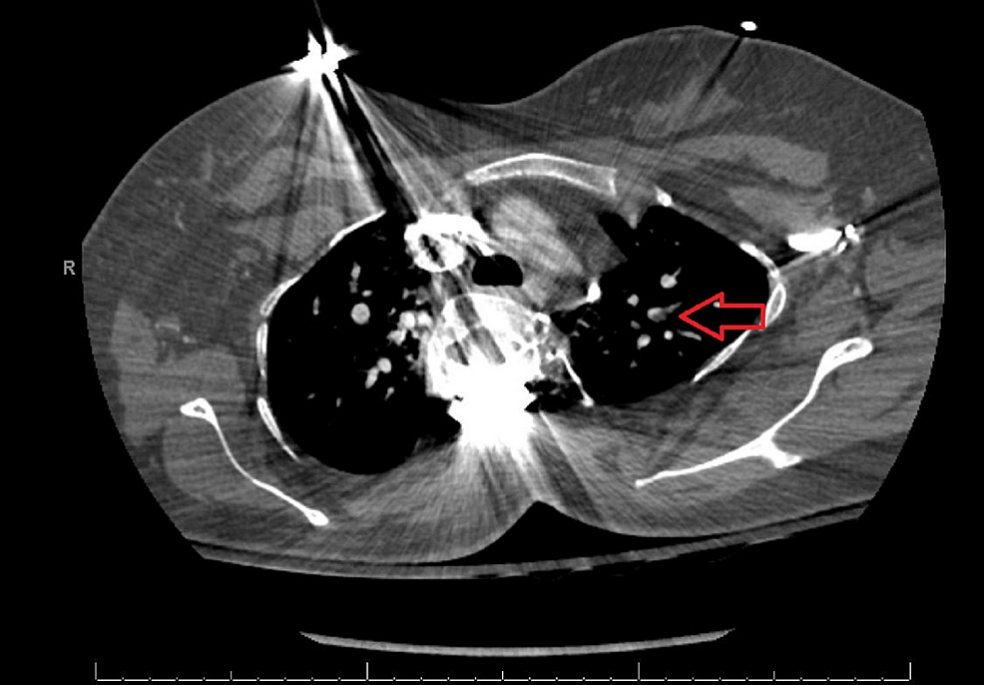
CT pulmonary angiography. Red arrow: evidence of a clot in the left upper subsegmental pulmonary artery branch. CT: computed tomography

On presentation, the patient was hypertensive with an oxygen saturation of 68% on room air, moderate pulmonary vascular congestion on the chest X-ray (Figure 1), and acute respiratory failure with an arterial carbon dioxide partial pressure of 69 mmHg. Further evaluation revealed a pulmonary embolus involving a left upper lobe sub-segmental pulmonary artery branch without right heart strain (Figure 2), for which anticoagulation with a heparin infusion was initiated. She was transferred to the Intensive Care Unit (ICU) for further monitoring and supportive care. During this time, her blood pressure remained elevated and her respiratory status continued to decline. She was given supplemental oxygen, and then transitioned to non-invasive ventilation (NIV), with settings of inspiratory positive airway pressure (iPAP) of 25 cmH^2^O and expiratory positive airway pressure (ePAP) of 5 cmH_2_O. She was diagnosed with preeclampsia with severe features and was prepared for an urgent cesarean section. The patient received betamethasone for fetal lung maturity and diuretics with furosemide for pulmonary edema. Her heparin infusion was held for four hours to reduce the complications related to hemorrhage in the setting of anticoagulation. An echocardiogram showed normal left and right-sided ventricular functions with no evidence of valvular abnormalities.

**Figure 3:**
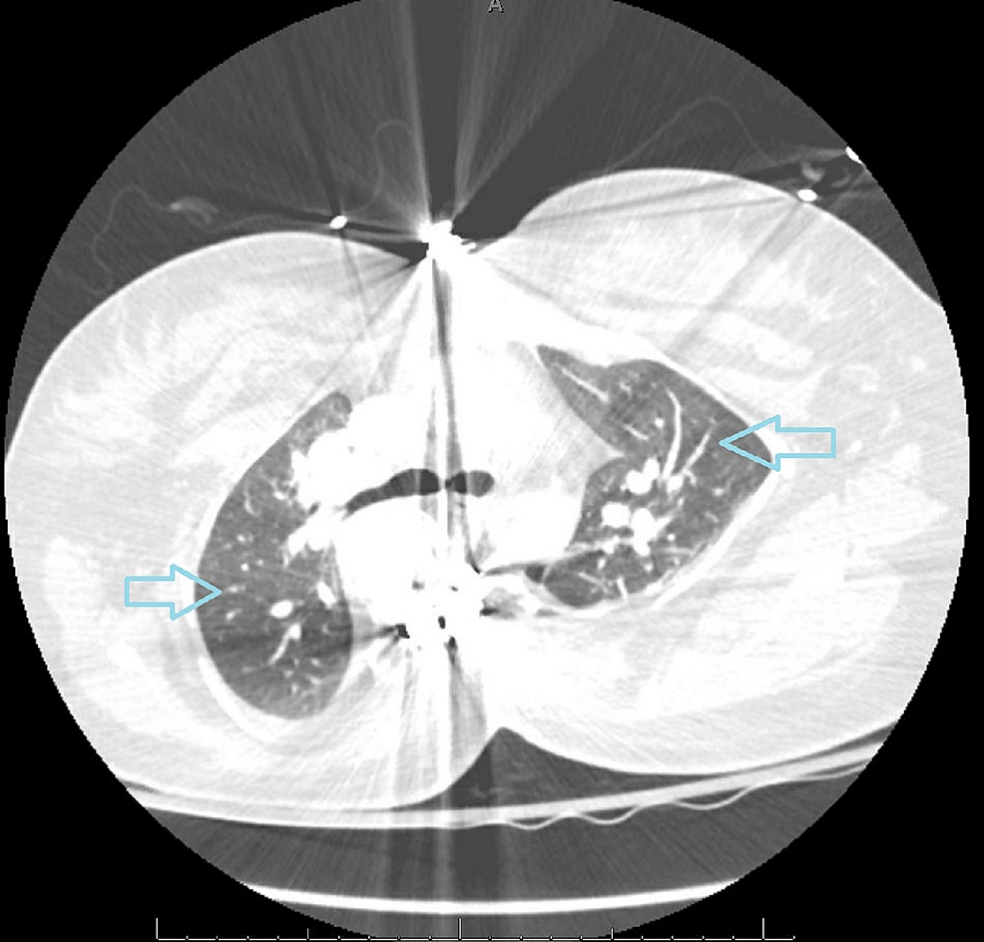
CT of the chest, lung window. Blue arrows: evidence of restrictive lung disease with decreased lung volumes. CT: computed tomography

Physical examination revealed an ill-appearing female in acute respiratory distress with an NIV mask. Her airway examination was notable for a Mallampati grade IV, two finger-breadth thyromental distance, limited mouth opening, and a high-arched palate. Her neck range of motion was unremarkable. She had two large-bore peripheral intravenous and an arterial line.

The delivery was scheduled in the operating room suite. The anesthesia machine was prepared with malignant hyperthermia precautions. This included disconnecting all volatile agents, flushing the anesthesia circuit with 10 L per minute oxygen flow through the circuit for over 20 minutes, and replacing the CO_2_ absorbent with a new unit. A cooling blanket was also placed on the operating room table. We prepared multiple airway devices including a flexible fiberoptic bronchoscope, laryngeal mask airways of varying sizes, an emergency cricothyrotomy kit, a jet ventilator, and an open surgical tracheostomy tray. The on-call trauma surgeon was on standby to complete a surgical airway if needed.

A multidisciplinary team was present in the operating room, which included the anesthesiologist, obstetrician, trauma surgeon, neonatologist, and nursing team. Upon entry to the operating room, the patient was transferred to the operating room table. Her NIV machine was disconnected, and she was given a simple mask with pressure support of 25 cmH_2_O. She was further positioned, and the surgical site was prepped and draped for immediate incision after induction of anesthesia. The patient’s neck was exposed but not prepped for a potential surgical airway.

She was safely induced using an awake video laryngoscope technique. She was administered 0.2 mg glycopyrrolate to decrease her respiratory secretions. Next, we anesthetized her oropharynx with viscous 4% lidocaine. We tested her glossopharyngeal nerve using a tongue blade. She tolerated the insertion of a size three video laryngoscope blade well, and we used an atomizer with additional local anesthetic as needed. A 6.0 endotracheal tube was easily placed and confirmed on both a physical examination and continuous end-tidal CO_2_. The patient was then quickly induced with propofol and fentanyl.

As soon as the airway was secured, the obstetrical team performed a cesarean section. A live male neonate weighing 1,135 g was delivered with APGAR scores of 7 at one minute and 9 at five minutes. Hemostasis was readily achieved. Anesthesia was maintained using propofol infusion at a rate of 100-150 µg/kg/minute in addition to 20 mg of ketamine bolus. Muscle relaxant was not used. The rest of the surgical course was unremarkable. After surgical closure, the patient was transferred to the ICU, intubated, and sedated. She was extubated the following day.

Postoperatively, the patient continued to have issues with hypoxia and hypercarbia. She was transitioned from heparin infusion to daily enoxaparin injections as she decided to breastfeed her baby. She has continued to require a home nasal cannula of 2 LPM oxygen during the daytime. At nighttime, she uses an NIV machine with a setting of 25 cmH_2_O iPAP and 5 cmH_2_O ePAP for her hypercarbia. She uses an albuterol inhaler as needed. She is under follow-up with the pulmonary clinic, which has appreciated both chronic restrictive and obstructive pulmonary disease (Table 1). She is still undergoing outpatient treatment to optimize her respiratory status.

**Table 1:** Findings of the pulmonary function test. FVC: forced vital capacity; FEV1: forced expiratory volume in the first second; DLCO: diffusion capacity for carbon monoxide; VA: alveolar volume

	Result	Reference	% of reference
Spirometry			
FVC	0.82 L	3.26 L	25%
FEV_1_	0.60 L	2.74 L	22%
FEV_1_/FVC	72%	84%	
Diffusion capacity			
DLCO	8.0 mL/mmHg/minute	22.9 mL/mmHg/minute	35%
DLCO/VA	5.7 mL/mmHg/minute	5.0 mL/mmHg/minute	113%

## Discussion

There were many anesthetic challenges involved with this case. In an emergent situation, we needed to optimize her compromised cardiopulmonary disorder. Her heparin infusion was held to control surgical bleeding. In the setting of hypertension and tenuous blood pressure readings, we utilized an arterial line. This allowed for repeated arterial blood gas sampling to monitor her respiratory status. In addition, we had two large-bore peripheral intravenous lines to ensure rapid delivery of fluids and blood products if needed.

While the patient’s history of thoracolumbar Harrington rods did not contradict the use of neuraxial anesthesia, the neuraxial spread of local anesthesia was more likely to be poor. There was a high chance of failure of the epidural to function optimally during delivery. If this were to occur, with any possible coinciding maternal respiratory distress or fetal distress, then an emergent airway would have been needed and the outcome would be potentially catastrophic. In addition, our patient needed heparin infusion to treat her pulmonary embolism, and neuraxial anesthesia might delay restarting heparin infusion. We chose to safely secure the patient’s airway as she was awake and spontaneously breathing and used general anesthesia with an endotracheal tube. This allowed us to minimize the potential need for an emergent airway in an uncontrolled state.

One of the most challenging aspects of completing awake intubations is delivering an anesthetic that allows for spontaneous ventilation and sedation. Our key to success was keeping our induction simple and communicating effectively with our patient. We talked extensively to our patient preoperatively that awake intubation would be the safest for her, and discussed what she might feel, hear, and experience during this technique. Though there are multiple ways to anesthetize the airway for awake intubation including inhalation, induction with sevoflurane is frequently employed for intubation of patients with a difficult airway, but this was contraindicated in our patient because of the risk of malignant hyperthermia [5-9].

A multidisciplinary approach is needed when patients have multiple complex issues such as FSS with preeclampsia, pulmonary edema, and a pulmonary embolus. After a discussion with the obstetrics team, we decided to keep our patient intubated. We felt that there would be a risk of postoperative respiratory compromise given her pulmonary embolus and increasing NIV settings. We did not feel these would resolve immediately after delivery. She was safely extubated the following day. However, she has continued to require home oxygen and nighttime NIV for six months after delivery. Postoperatively, the patient continued to have issues with hypoxia and hypercarbia. She was transitioned from heparin infusion to daily enoxaparin injections as she decided to breastfeed her baby [11]. The patient received daily enoxaparin injections as there is some evidence that oral anticoagulants might be excreted in breast milk [11].

## Conclusions

Adequate knowledge of the anesthetic considerations of the physical and metabolic characteristics of FSS is important when developing an anesthetic plan. This involves mainly taking into account a difficult airway, underlying restrictive lung disease, and bone fragility. Moreover, the risk of malignant hyperthermia should be taken into consideration when dealing with these patients.
